# Tauroursodeoxycholic Acid Inhibited Apoptosis and Oxidative Stress in H_2_O_2_-Induced BMSC Death via Modulating the Nrf-2 Signaling Pathway: the Therapeutic Implications in a Rat Model of Spinal Cord Injury

**DOI:** 10.1007/s12035-023-03754-5

**Published:** 2023-11-28

**Authors:** Jiaxian Weng, Le Wang, Kai Wang, Haitao Su, Dan Luo, Haimei Yang, Yaqian Wen, Qiduan Wu, Xing Li

**Affiliations:** 1 School of Pharmaceutical Sciences, Guangzhou University of Chinese Medicine, Guangzhou, 510006 Guangdong China; 2 Lingnan Medical Research Center of Guangzhou University of Chinese Medicine, Guangzhou, 510405 China; 3 Guangzhou University of Chinese Medicine, Guangzhou, 510405 China; 4 Department of Spine Surgery, the First Affiliated Hospital of Sun Yat-Sen University, Guangdong Provincial Key Laboratory of Orthopedics and Traumatology, Guangzhou, 510080 Guangdong China; 5 State Key Laboratory of Dampness Syndrome of Chinese Medicine, Department of Orthopedic Surgery,, The Second Affiliated Hospital of Guangzhou University of Chinese Medicine, Guangzhou, 510120 China; 6 The First Affiliated Hospital of Chinese Medicine, Guangzhou University of Chinese Medicine, Guangzhou, 510405 China

**Keywords:** Bone marrow mesenchymal stem cells, TUDCA, Spinal cord injury, Apoptosis, Oxidative stress

## Abstract

Spinal cord injury (SCI) is a prevalent and significant injury to the central nervous system, resulting in severe consequences. This injury is characterized by motor, sensory, and excretory dysfunctions below the affected spinal segment. Transplantation of bone marrow mesenchymal stem cells (BMSCs) has emerged as a potential treatment for SCI. However, the low survival as well as the differentiation rates of BMSCs within the spinal cord microenvironment significantly limit their therapeutic efficiency. Tauroursodeoxycholic acid (TUDCA), an active ingredient found in bear bile, has demonstrated its neuroprotective, antioxidant, and antiapoptotic effects on SCI. Thus, the present study was aimed to study the possible benefits of combining TUDCA with BMSC transplantation using an animal model of SCI. The results showed that TUDCA significantly enhanced BMSC viability and reduced apoptosis (assessed by Annexin V-FITC, TUNEL, Bax, Bcl-2, and Caspase-3) as well as oxidative stress (assessed by ROS, GSH, SOD, and MDA) both in vitro and in vivo. Additionally, TUDCA accelerated tissue regeneration (assessed by HE, Nissl, MAP2, MBP, TUJ1, and GFAP) and improved functional recovery (assessed by BBB score) following BMSC transplantation in SCI. These effects were mediated via the Nrf-2 signaling pathway, as evidenced by the upregulation of Nrf-2, NQO-1, and HO-1 expression levels. Overall, these results indicate that TUDCA could serve as a valuable adjunct to BMSC transplantation therapy for SCI, potentially enhancing its therapeutic efficacy.

## Introduction

Spinal cord injury (SCI) is a prevalent trauma to the central nervous system with serious consequences [1]. It is typically characterized by motor, sensory, and excretory dysfunctions below the level of the injury, which can result in permanent disability or even death, depending on the extent and severity of the injury. In 40% of cases, it leads to complete tetraplegia due to motor and sensory along with excretory dysfunction below the site of injury. The costs associated with caring for SCI patients can reach as high as $100,000 per year, imposing a significant financial burden on patients and their families [2, 3]. Consequently, SCI greatly affects patients’ daily lives, causing physical and psychological harm, along with an economic burden. The pathophysiological process of SCI is composed of primary injury as well as secondary damage [4]. Primary injuries, such as stroke, compression, and torsion, lead to numerous neuronal cell necrosis within a brief time interval and generate a large amount of reactive oxygen species (ROS) in the injured tissue [5–7]. Moreover, the secondary injury is primarily associated with the heightened generation of initial ROS components, accompanied by cellular lipid peroxidation cascade [6, 8]. Oxidative stress (OS), as a critical element of secondary injury, can disrupt cell membrane integrity and eventually result in cell necrosis [9]. Thus, controlling OS in secondary SCI would be a crucial breakthrough in SCI treatment.

To address SCI, researchers have explored the potential of stem cell–based therapeutic interventions, specifically focusing on bone marrow mesenchymal stem cell (BMSC) therapy [10–12]. BMSCs, abundant in the bone marrow, possess the multi-directional differentiation ability into nerve cells, making them suitable for filling the damaged site and promoting tissue regeneration after SCI [10, 13]. Transplantation of BMSCs into an SCI mouse model has demonstrated both histological and functional recovery [14, 15]. However, the therapeutic potential of BMSCs in SCI treatment is hindered by their low survival and differentiation rates within the spinal cord microenvironment. These limitations present a significant challenge in translating BMSCs into a clinically feasible treatment alternative for SCI [16, 17]. The key factors contributing to this limitation are directly associated with the OS response in the SCI area [16, 18, 19]. Reducing OS is crucial for preserving the survival rate of transplanted BMSCs, as excessive oxidative reactions can significantly impede their viability following transplantation. Therefore, within the context of SCI, strategies focused on alleviating OS may potentially enhance the effectiveness of BMSC transplantation.

Tauroursodeoxycholic acid (TUDCA) is a bioactive compound found in the bile of various bear species. It has demonstrated pharmacological characteristics such as anti-oxidation, anti-inflammation, and anti-apoptosis, as well as neuroprotection [20–23]. TUDCA can traverse the blood–brain barrier without detrimental effects and has been shown to enhance axon regeneration and improve motor function recovery in models of SCI [20, 24]. Combining TUDCA with BMSC transplantation holds significant potential for SCI treatment. However, the precise mechanisms underlying this combined therapy remain elusive. Therefore, we aimed to assess the value of TUDCA in ameliorating OS-mediated BMSC apoptosis while investigating the underlying mechanisms in the present study. Additionally, we examined the efficacy of combining TUDCA with BMSC transplantation in SCI rats to evaluate functional recovery. Our results may have significant implications for the clinical translation of TUDCA as an adjunct therapeutic agent in conjunction with BMSC transplantation in the management of SCI.

## Materials and Methods

### Materials

Male Sprague Dawley (SD) rats were obtained from the Experimental Animal Center at the Guangzhou University of Chinese Medicine in Guangzhou, China. All animal experiments were carried out following the guidelines established by the Guangzhou University of Chinese Medicine and approved by its ethics committee. TUDCA, (purity > 98%) was obtained from Shanghai Yuanye Bio-Technology Company Limited, located in Shanghai, China.

### Isolation, Culture, and Treatment of BMSCs

The purity of BMSCs derived from male SD rats (2-week-old) was assessed complying with the methodology previously outlined [25]. BMSCs from passages 3 to 5 were cultured in α-modified Eagle’s medium supplemented with 10% fetal bovine serum, under standard cell culture conditions of 37 °C and 5% CO_2_, with the culture medium changed every other day. Before H_2_O_2_ treatment, cells were pretreated with TUDCA for 2 h.

### Cell Viability (CV) Assay

To assess CV, BMSCs were cultured in 96-well plates with a density of 1 × 10^4^ cells/well) for 24 h. Subsequently, the BMSCs (either alone or in combination with TUDCA) were treated with H_2_O_2_ for another 24 h. The cell counting kit-8 (CCK-8, Biosharp, China) assay was performed according to the manufacturer’s instructions. Briefly, a CCK-8 solution (10 μL) was added to each well, followed by incubation at 37 °C for 2 h. The absorbance of each well was measured at 450 nm using a microplate reader (Bio-Rad) to determine CV. All of these experiments were triply conducted.

### Flow Cytometric Evaluation of Apoptosis

To assess the apoptosis rate of BMSCs in vitro, an Annexin V-FITC apoptosis detection kit (Invitrogen, USA) was used, following the manufacturer’s instructions. BMSCs were collected, gently washed using phosphate-buffered saline (PBS), and suspended in binding buffer (500 μL), supplemented with Annexin V-FITC (5 μL) as well as propidium iodide (10 μL). Following a 10-min incubation at room temperature, the samples were subjected to flow cytometry analysis (BD Biosciences).

### Terminal Deoxynucleotidyl Transferase dUTP Nick End Labeling (TUNEL) Assay

The quantification of apoptotic cells was achieved by TUNEL staining utilizing a commercially available cell apoptosis detection kit (Yeasen Biotech, Shanghai, China) and quantified by capturing immunofluorescent images with a fluorescence microscope (Olympus IX73).

### ROS Assay

To detect cellular levels of ROS, the dichloro-dihydro-fluorescein diacetate (DCFH-DA) staining kit (Jiancheng Institute of Biology, Nanjing, China) was used according to the manufacturer’s instructions. Following staining, a fluorescence microscope (Olympus IX73) was utilized to visualize the stained cells.

### Glutathione (GSH), Superoxide Dismutase (SOD), and Malondialdehyde (MDA) Measurement

To measure GSH, SOD, and MDA levels, commercial kits were used complying with the instructions provided by the corresponding manufacturers. GSH absorbance was measured at 412 nm, SOD activity at 560 nm, and MDA levels at 532 and 600 nm. All experiments were conducted in triplicate to ensure the accuracy and reproducibility of the results.

### Establishment of SCI Model and Treatment

Adult male SD rats (200–250 g) were allocated randomly into 5 groups: sham-operated, SCI, SCI + BMSCs, SCI + TUDCA, as well as SCI + BMSCs + TUDCA (*n* = 12 per group). The surgical procedure involved the intraperitoneal administration of sodium pentobarbital (40 mg/kg) to induce anesthesia. Following the anesthesia, spinal cord laminectomy was performed at the T9–T10 vertebral level in adult male SD rats to expose the spinal cord. SCI was induced in adult male SD rats using a pneumatic impact device with a force of 1.2 m/s, a constant depth of 1.0 mm, as well as a duration of 85 ms. The injury was confirmed by spinal cord congestion, leg swaying, reflexes of tail swing, and gradual paralysis. Rat bladders were manually emptied twice daily until normal urination was possible. Sham-operated rats underwent surgical procedures without SCI. Rats in the BMSCs and SCI + BMSCs + TUDCA groups received BMSCs through the tail vein 3 days after SCI induction. TUDCA (200 mg/kg, oral administration) was fed once a day following injury according to previous reports [20]. Rats in vehicle groups underwent similar procedures and treatments. After completing the treatment, spinal cords were harvested and stored at − 80 ℃ until further analysis.

### Hematoxylin and Eosin (H&E) and Nissl Staining

Under anesthesia after saline perfusion, the rats were perfused with paraformaldehyde (4%) in PBS (0.1 M, pH 7.4) through the heart to preserve their structural integrity. After fixation with 4% paraformaldehyde, the harvested spinal cord tissues were embedded in paraffin and sectioned (5 μm in thickness) for subsequent analysis. After overnight baking at a temperature of 37 ℃, sections were subjected to H&E and Nissl staining according to the manufacturer’s instructions.

### Immunofluorescence Staining and Analysis

According to a previous study [20], the tissues were subjected to overnight incubation at the temperature of 4 °C with microtubule-associated protein 2 (MAP2) (1:200, Boster Biological Engineering Co.), glial scar formation (GFAP) (1:200, Boster Biological Engineering Co.), myelin basic protein (MBP) (1:200, Boster Biological Engineering Co.), and beta-tubulin III (TUJ1) (1:200, CST) as primary antibodies. The following day, the tissues were incubated with Alexa Fluor (1:300, Invitrogen) diluted in PBS according to the corresponding primary antibodies, and nuclei were stained with 4′,6-diamidino-2-phenylindole (DAPI). Fluorescent images were viewed under a fluorescence microscope.

### Western Blotting (WB) Analysis

Protein samples from spinal cord tissues and cells were extracted using ristocetin-induced platelet aggregation buffer (Beyotime, Shanghai, China) and quantified using a bicinchoninic acid protein assay kit (Beyotime, Shanghai, China). Following separation by 10% sodium dodecyl sulfate–polyacrylamide gel electrophoresis (SDS-PAGE), the samples were transferred to a polyvinylidene fluoride (PVDF) membrane. To prevent nonspecific binding, the membrane was blocked with 5% skim milk for a duration of 1 h at ambient temperature before incubating with primary antibodies against Bax (1:1000, CST), Bcl-2 (1:1000, Abcam), Caspase-3 (1:1000, CST), nuclear factor E2-related factor 2 (Nrf-2) (1:1000, Affinity Bioscience), NAD(P)H: quinone oxidoreductase-1 (NQO-1) (1:1000, Abcam), and Heme Oxygenase-1 (HO-1) (1:1000, Abcam) overnight at 4 °C. The protein levels were normalized against the reference protein, glyceraldehyde-3-phosphate dehydrogenase (1:1000, Abcam). Following TBST (tris buffered saline + 0.5% [v/v] Tween-20) washing, the membrane was incubated with horseradish peroxidase–conjugated secondary antibodies for 1 h. Signals were visualized using the ChemiDocTM MP Imaging System (Bio-Rad) and quantified with ImageJ software (National Institutes of Health, Bethesda, MD).

### Quantitative Real-Time Polymerase Chain Reaction (qRT-PCR) Analysis

Total RNA extraction from the BMSCs was performed using TRIzol reagent (Invitrogen, USA) complying with the instructions provided by the manufacturer. The cDNA synthesis was performed using the PrimeScript RT kit (Takara Biotechnology, Dalian, China). Besides, the SYBR Green Premix Pro Taq HS qPCR kit II (Accurate Biotechnology, Guangzhou, China) was utilized in qRT-PCR. The primer sequences used in the experiment are listed in Table 1.

**Table 1:** Primers used for target amplification in this study

Name	Primer	Sequence (5′-3′)
Bcl-2	Forward	CTGTGGATGACTGAGTACCTGAAC
	Reverse	AGAGACAGCCAGGAGAAATCAAAC
Bax	Forward	CACCAGCTCTGAACAGATCA
	Reverse	ATCGCCAATTCGCCTGAGAC
GAPDH	Forward	CAAGTTCAACGGCACAGTCAAG
	Reverse	ACATACTCAGCACCAGCATCAC

### Behavioral Assessment

Hindlimb locomotor function post-SCI was assessed on days 1, 7, 14, 21, and 28 using the Basso, Beattie, and Bresnahan (BBB) locomotor test. This test evaluated joint movements, coordination, stepping ability, and trunk stability. The test scores were measured on a scale of 0 to 21, with a score of 21 representing normal motor function in sham-operated rats.

On day 28 post-SCI, these rats underwent the footprint test, which involved walking in a straight line down a narrow path while their footprints were recorded. The footprints were then analyzed digitally to evaluate gait.

### Statistical Analysis

Data were analyzed using SPSS 24.0 (SPSS Inc., USA) and presented as mean ± standard deviation (SD). Meanwhile, one-way analysis of variance (ANOVA) and Student’s *t*-test were utilized to compare the differences between groups. *P* value < 0.05 indicated statistical significance.

## Results

### Protective Effects of TUDCA on H_2_O_2_-Induced BMSCs

**Fig. 1:**
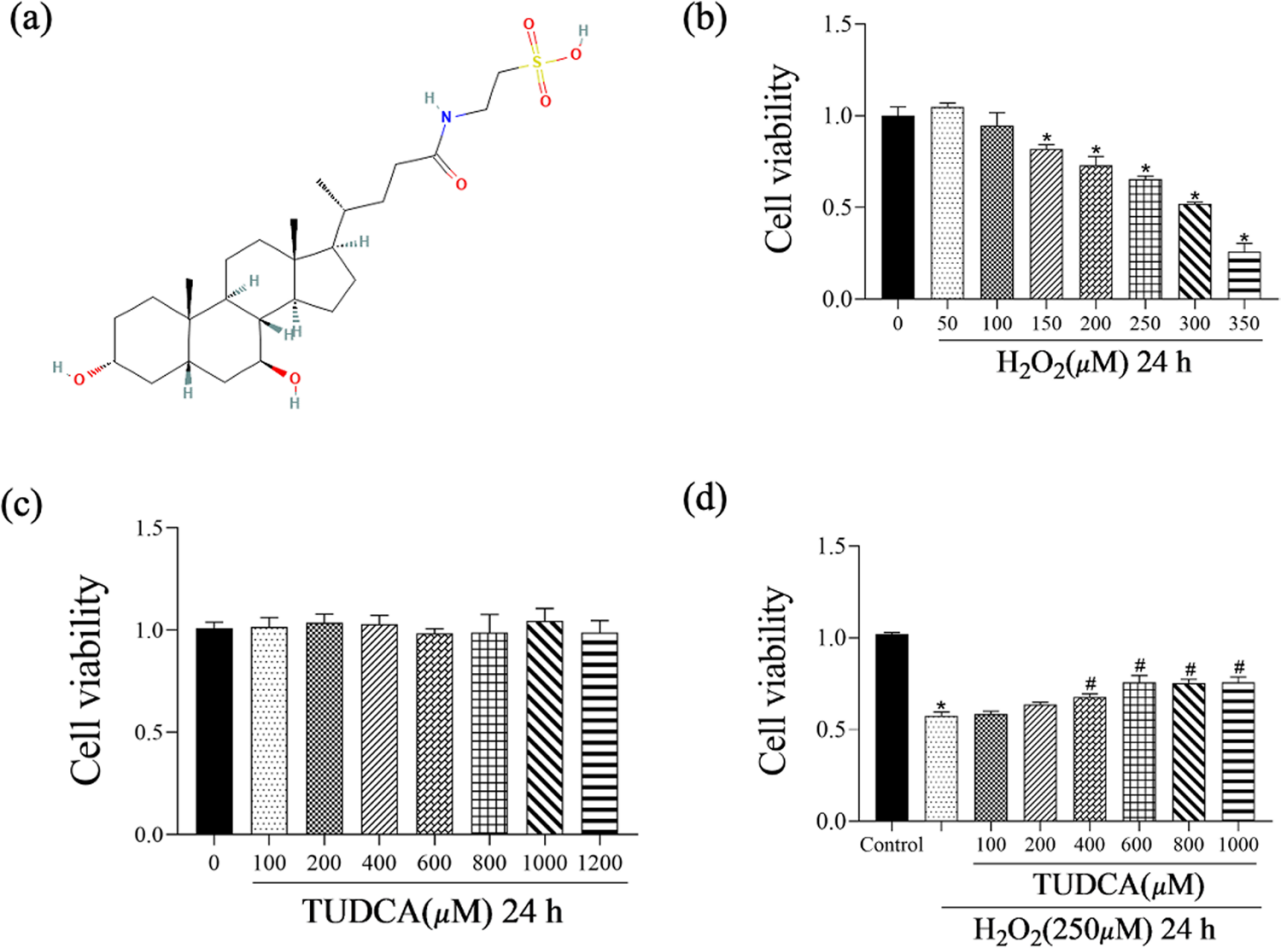
Effects of TUDCA on the viability of H_2_O_2_-induced BMSCs. Cells were treated with different concentrations of TUDCA (100–1200 μM) and H_2_O_2_ (250 μM) for 24 h. **a** Structure of TUDCA. **b** Cells are cultured with different concentrations of H_2_O_2_ (50–350 μM) for 24 h. **c** BMSC viability with/without TUDCA treatment following 24 h of culture. **d** Viability of H_2_O_2_-induced BMSCs treated with various concentrations of TUDCA. Data are presented as mean ± SD (*n* = 3); ^*^P < 0.05 vs. the control group. ^#^*P* < 0:05 vs. the group with H_2_O_2_-induced alone

CV of BMSCs decreased in a concentration-dependent manner following 24 h of H_2_O_2_ induction with statistical significance. Within the tested range, H_2_O_2_ concentration in 250 μM caused approximately 60–70% cell death (Fig. 1b). However, BMSCs exposed to TUDCA for 24 h did not exhibit any cytotoxic effects, maintaining constant CV at all concentrations tested from 0 to 1200 μM (Fig. 1c). CCK-8 assays confirmed that TUDCA significantly increased CV (Fig. 1d). Based on these results, the concentration of TUDCA used in subsequent experiments was chosen as 600 μM.

### TUDCA Alleviated H_2_O_2_-Induced Apoptotic BMSC Cells

**Fig. 2:**
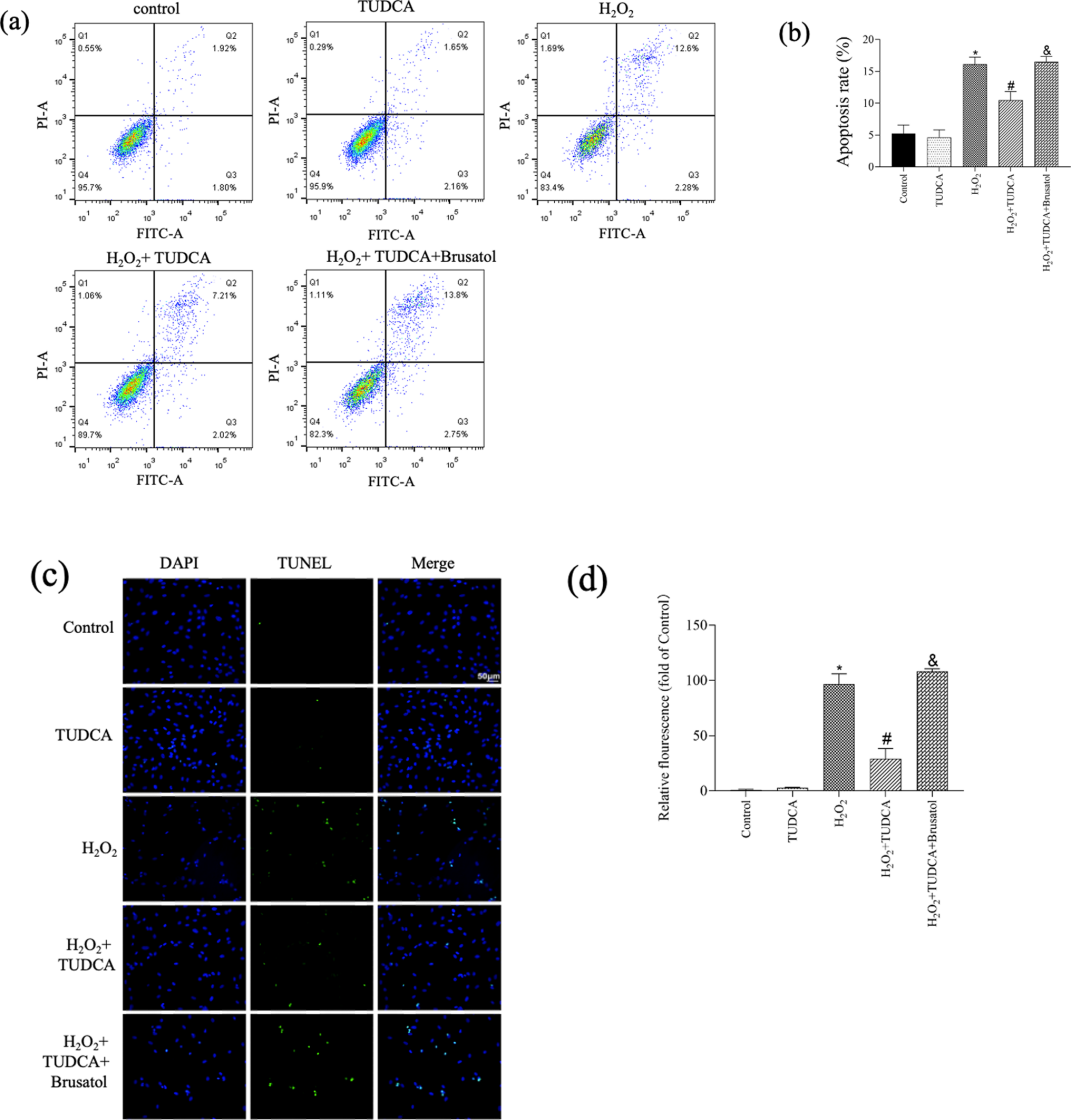

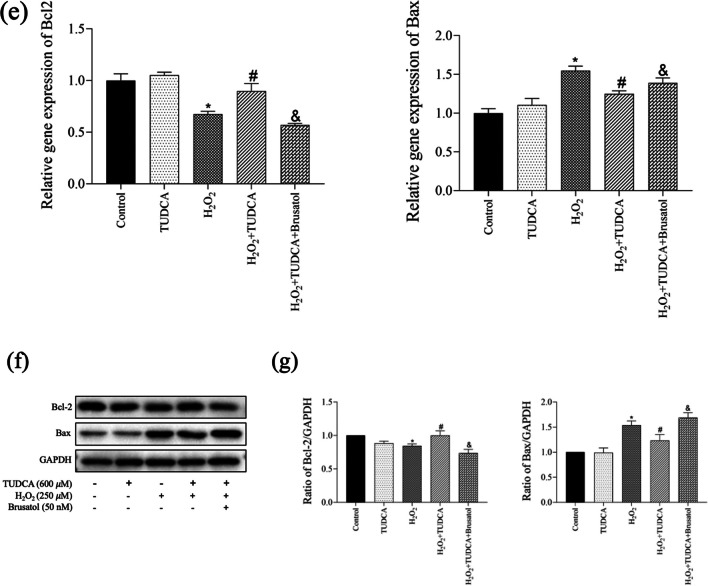
TUDCA attenuated H_2_O_2_-induced apoptosis in BMSCs. Cells were treated with TUDCA (600 μM) and H_2_O_2_ (250 μM) for 24 h. **a**, **b** Evaluation of cell apoptosis using Annexin V-FITC/PI assay. **c**, **d** Analysis of cell apoptosis using TUNEL staining. **e** Detection of Bcl-2 and Bax gene expressions using real-time quantitative PCR. **f**, **g** Measurement of Bcl-2 and Bax protein expressions using Western blot. Data are presented as mean ± SD (*n* = 3); ^*^P < 0.05 vs. the control group. ^#^*P* < 0:05 vs. the H_2_O_2_-induced alone group. ^&^*P* < 0.05 vs. the TUDCA + H_2_O_2_-induced group

To study whether TUDCA could protect BMSCs from the apoptosis induced by H_2_O_2_, several assays were performed, including Annexin V/PI staining, TUNEL assay, qRT-PCR, and WB analysis. Annexin V/PI staining revealed that the apoptotic rate was 3.72% ± 1.37 in the control group, and then it was increased to 14.88% ± 1.11 in the H_2_O_2_ group; however, it was reduced to 9.23% ± 1.33 in the H_2_O_2_ group pretreated with TUDCA (Fig. 2a and b). These findings were consistent with the TUNEL assay results (Fig. 2c and d). Analysis of antiapoptotic (Bcl-2) and proapoptotic (Bax) protein and mRNA levels demonstrated that H_2_O_2_ induction led to a decrease in Bcl-2 expression and an increase in Bax expression, which were significantly reversed by TUDCA treatment (Fig. 2e, f, and g). Notably, the protective role of TUDCA in the cell apoptosis induced by H_2_O_2_ was inhibited by Brusatol, an Nrf-2 signal inhibitor.

### Protective Effect of TUDCA on OS Among BMSCs

**Fig. 3:**
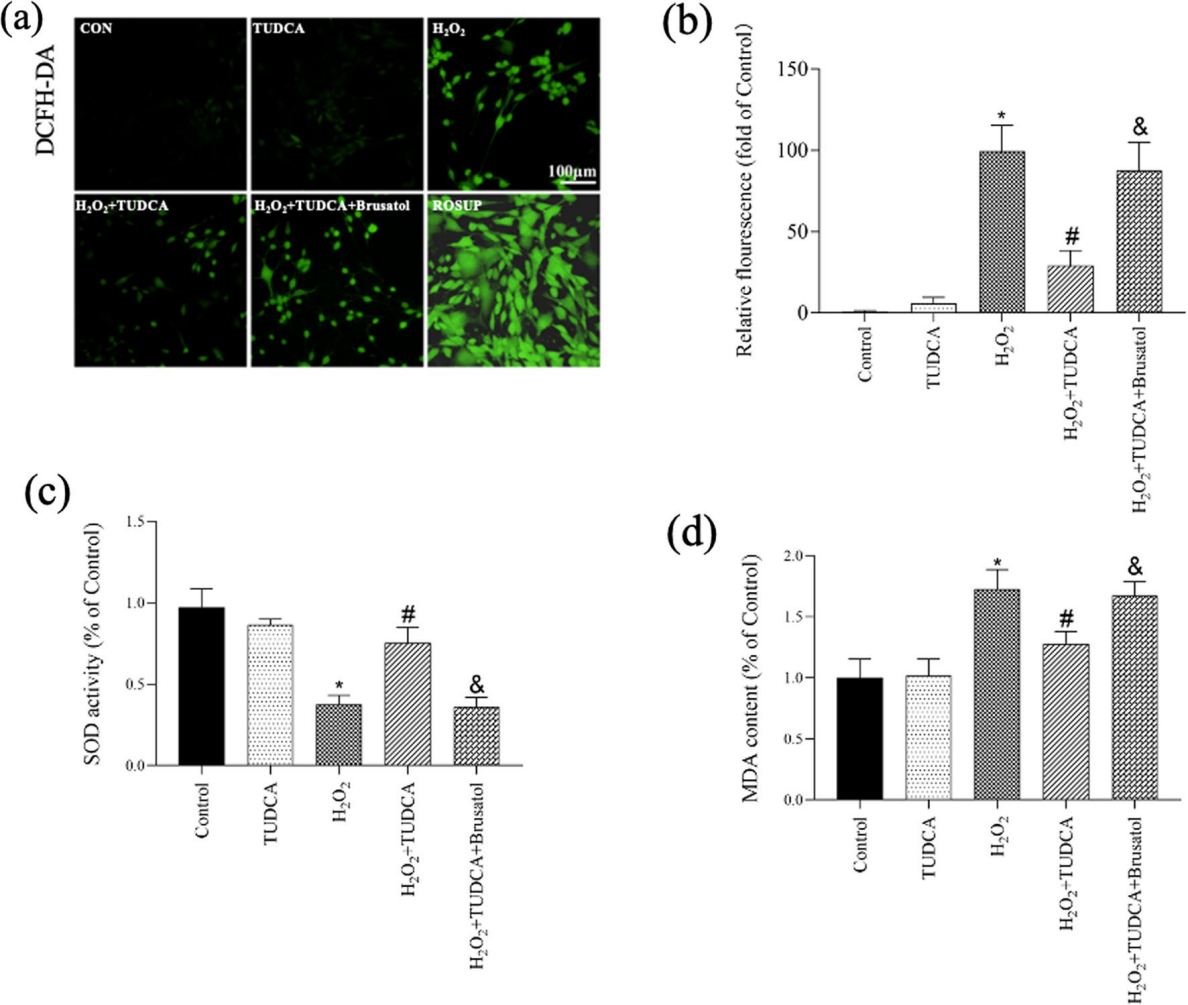
TUDCA treatment abrogated H_2_O_2_-induced oxidative stress. Cells were treated with TUDCA (600 μM) and H_2_O_2_ (250 μM) for 24 h. **a** Measurement of ROS production in BMSCs using DCFH-DA staining. **b** Quantitative analysis of DCF fluorescent intensity. **c** Measurement of SOD activity. **d** Measurement of MDA content. Data are presented as mean ± SD (*n* = 3); ^*^P < 0.05 vs. the control group. ^#^*P* < 0:05 vs. the H_2_O_2_-induced alone group. ^&^*P* < 0.05 vs. the TUDCA + H_2_O_2_-induced group

To confirm the protective effect of TUDCA on OS in BMSCs, we measured intracellular ROS levels (Fig. 3a, b), SOD activity (Fig. 3c), and MDA content (Fig. 3d). The H_2_O_2_-treated group exhibited a significant increase in ROS production compared to the control group, which was subsequently decreased by TUDCA treatment. Additionally, analysis of SOD activity and MDA content further confirmed the protective role of TUDCA in BMSCs against OS.

### TUDCA Inhibited H_2_O_2_-Induced Apoptosis and OS Within BMSCs Through Nrf-2 Signaling

**Fig. 4:**
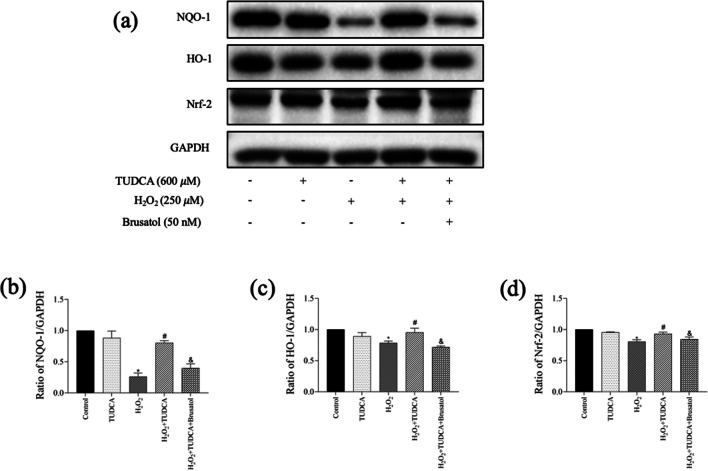
TUDCA protected BMSCs from H_2_O_2_-induced apoptosis and oxidative stress via the Nrf-2 signaling pathway. Cells were treated with TUDCA (600 μM) combined with H_2_O_2_ (250 μM) for 24 h. **a**, **b**, **c**, **d** Measurement of the protein expression of NQO-1, HO-1, and Nrf-2 by Western blot. Data are presented as mean ± SD (*n* = 3); ^*^P < 0.05 vs. the control group. ^#^*P* < 0:05 vs. the H_2_O_2_-induced alone group. ^&^*P* < 0.05 vs. the TUDCA + H_2_O_2_-induced group

We investigated the involvement of the Nrf-2 signaling pathway in mediating the protective effect of TUDCA against apoptosis as well as OS. WB analysis demonstrated the decreased NQO-1, HO-1, and Nrf-2 protein levels following H_2_O_2_ treatment with statistical significance. However, TUDCA treatment restored these protein levels (Fig. 4). Furthermore, partial blocking of TUDCA’s protective effect by Brusatol during H_2_O_2_-induced cytotoxicity was observed.

### TUDCA Accelerated Tissue Regeneration as well as Functional Recovery Following BMSC Transplantation in SCI

**Fig. 5:**
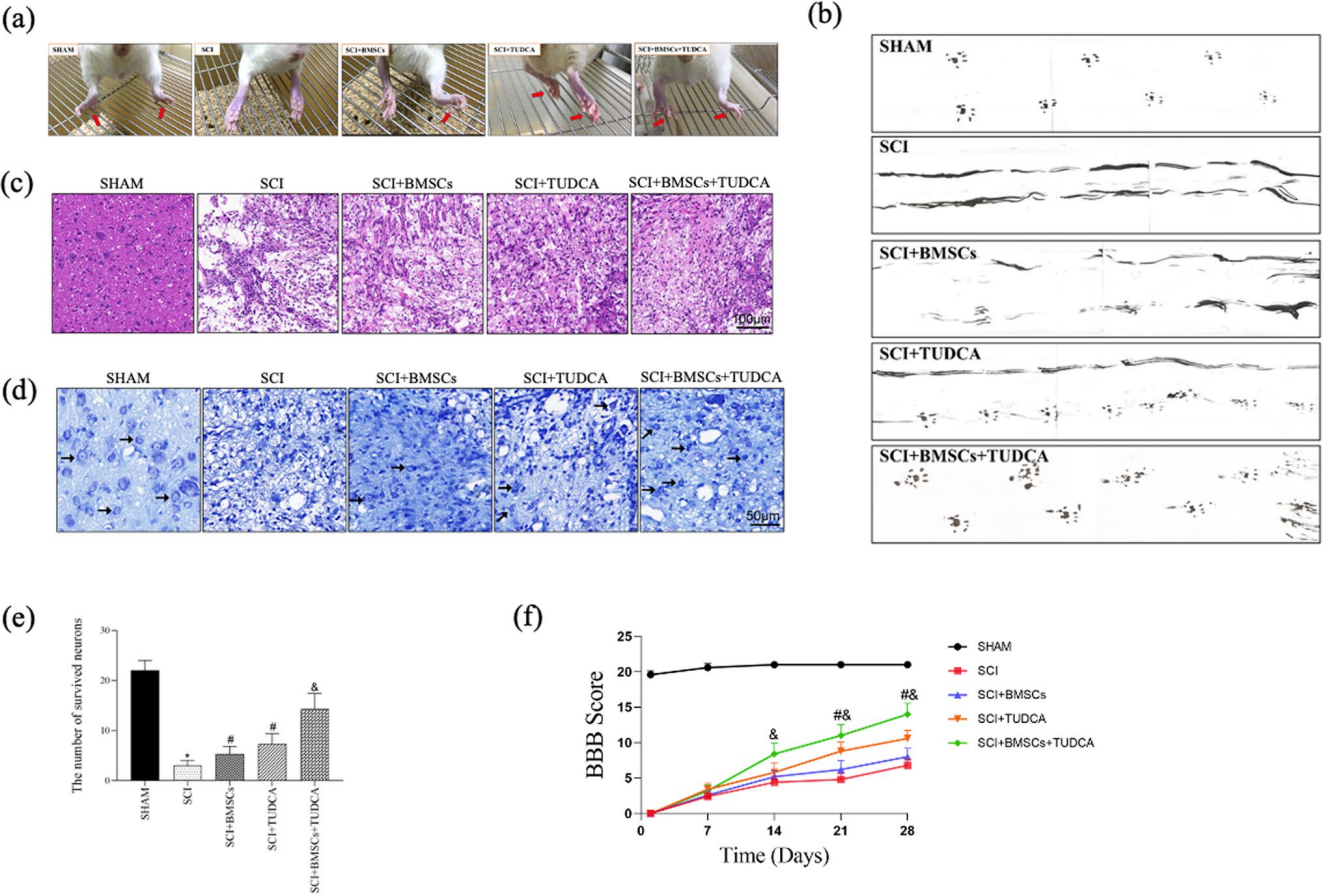

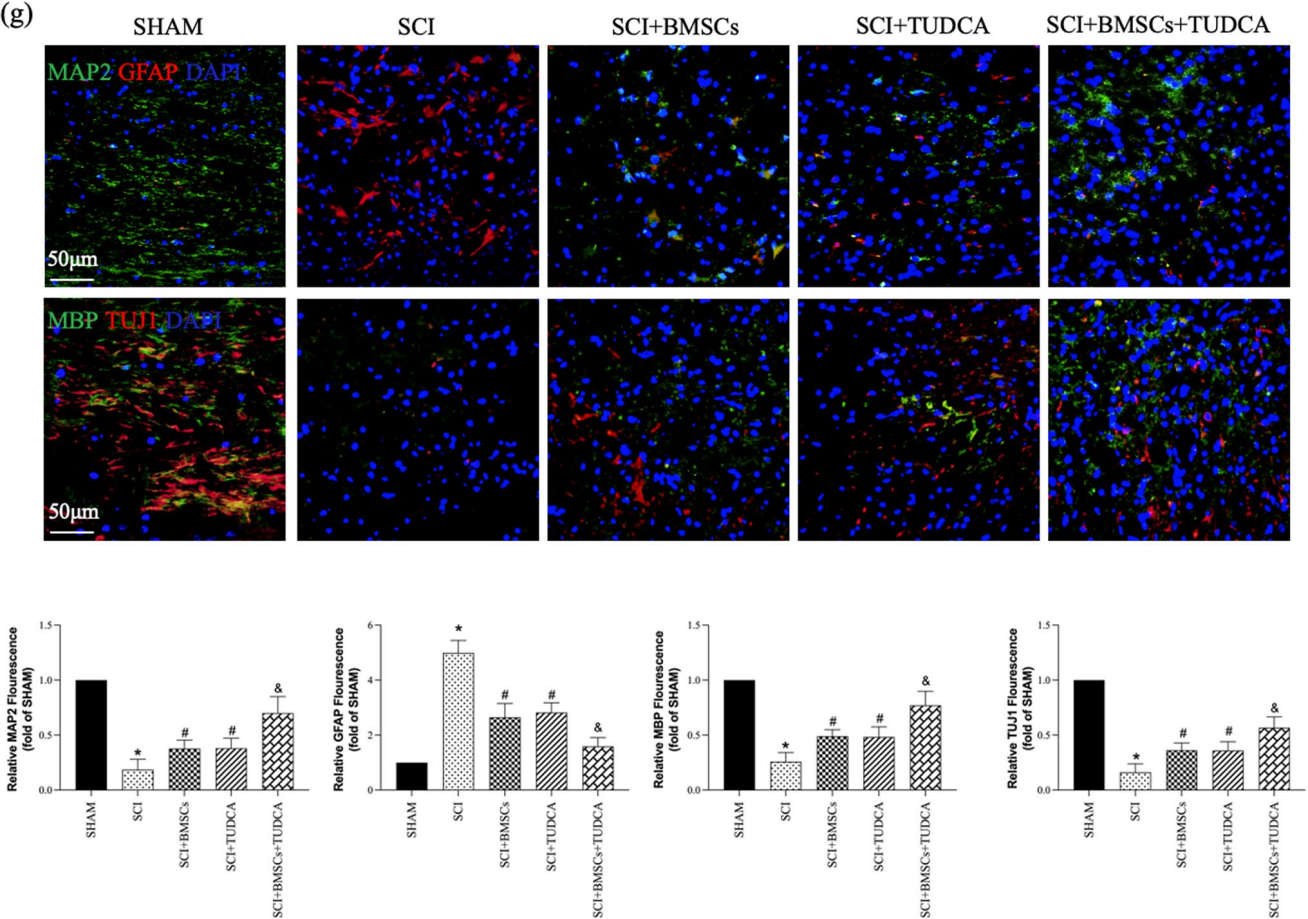
The combined treatment of TUDCA and BMSC transplantation accelerated the regeneration of tissues and motor functional recovery after SCI. **a** Images of behavioral observation showing the characteristics of hind limb movement of rats across different groups. **b** Footprint analyses of different groups on day 28 after SCI. **c** Representative images from H&E staining in longitudinal section on day 28 after SCI. **d**, **e** The survived neurons following Nissl staining and relative quantification at the lesion area from different groups on day 28 after SCI. **f** The Basso, Beattie, and Bresnahan (BBB) locomotor scores of different groups. **g** The Immunofluorescence staining of MAP2(green), MBP (green), TUJ1 (red), and GFAP (red) from different groups on day 28 post-SCI. Data are presented as mean ± SD. ^*^P < 0.05 vs. the SHAM group. ^#^*P* < 0:05 vs. the SCI group. ^&^*P* < 0.05 vs. the BMSC group (*n* = 6)

To explore the effect of TUDCA among SCI rats, a series of behavioral observations, footprint tests, HE staining, Nissl staining, and BBB scores were conducted to evaluate tissue regeneration and functional recovery. In the SCI group, the hindlimbs of the rats were weak, leading to impaired walking ability. However, TUDCA treatment significantly improved hindlimb function, as shown in Fig. 5a. Additionally, TUDCA administration in combination with BMSCs further enhanced hindlimb function in rats with SCI. The gait of rats after BMSC transplantation in SCI also improved with TUDCA treatment, resulting in relatively consistent hindlimb footprints and regained strength after 28 days (Fig. 5b).

Histological examination of spinal cords from SCI rats revealed deformities and cavities compared to the sham group. However, TUDCA together with the combined treatment reduced the lesion area and tissue damage compared to other SCI groups (Fig. 5c). Nissl staining demonstrated a significant increase in the neuron number within the lesions when TUDCA was combined with BMSC transplantation, surpassing other SCI groups (Fig. 5d, e). Immunofluorescence staining consistently demonstrated trends in the expression of MAP2, MBP, and TUJ1 (Fig. 5g).

Furthermore, BBB scores decreased at 1, 7, 14, 21, and 28 days after SCI in all groups except the Sham group. Despite that, the combination of TUDCA and BMSC transplantation improved BBB scores on day 14 post-SCI with statistical significance, showing higher scores on days 21 and 28 compared to other SCI groups (Fig. 5f). This combined treatment promoted tissue repair, neuronal survival, axonal regeneration, reduced glial scar formation, and facilitated functional recovery in SCI rats.

### Effects of BMSCs, TUDCA, Along with Their Combination on Apoptosis in SCI

**Fig. 6:**
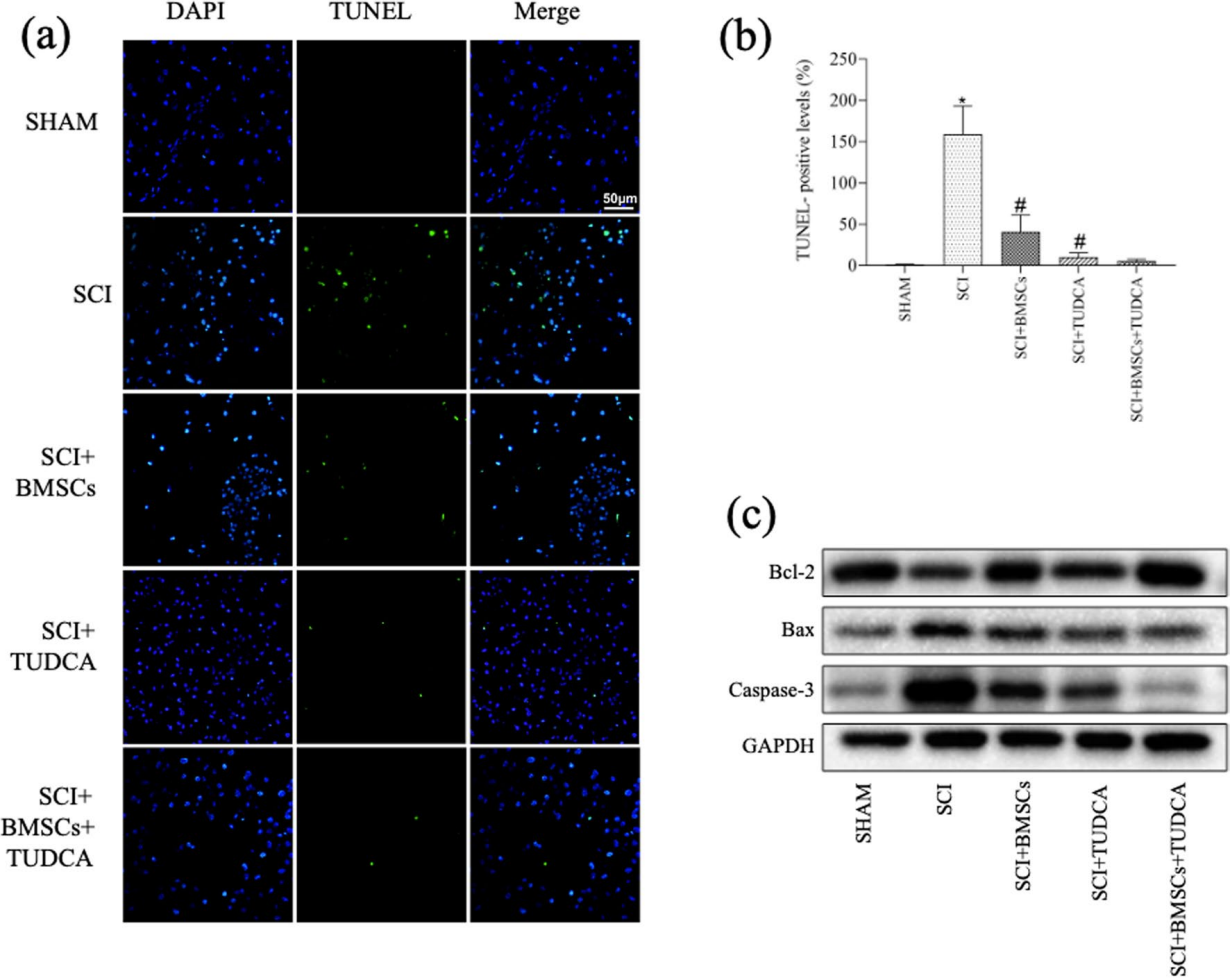

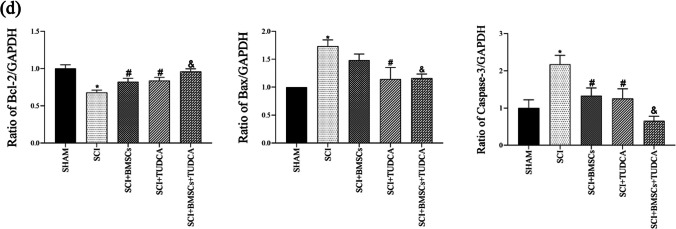
The combined treatment of TUDCA and BMSC transplantation reduced cell apoptosis in a rat SCI model. **a** TUNEL staining of neuronal apoptosis at 14 days post-SCI. **b** Quantitative estimation of the number of TUNEL-positive cells. **c** Western blot analysis showing Bcl-2, Bax, and Caspase-3 protein expressions in the samples of spine tissue. **d** Quantification of the relative Bcl-2, Bax, and Caspase-3 protein levels. Data are presented as mean ± SD. ^*^P < 0.05 vs. the SHAM group. ^#^*P* < 0:05 vs. the SCI group. ^&^*P* < 0.05 vs. the BMSC group (*n* = 6)

TUNEL staining revealed a significant increase of apoptotic cells following SCI, which was effectively reduced in the TUDCA and combined treatment groups in contrast with the SCI or SCI + BMSC groups (Fig. 6a, b). WB analysis confirmed the reduced Bcl-2 level as well as the increased levels of Bax and Caspase-3 in SCI. However, TUDCA combined with BMSC transplantation reversed these results (Fig. 6c, d), indicating a reduction in SCI-induced apoptosis.

### Effects of BMSCs, TUDCA, Along with Their Combination on OS in SCI

**Fig. 7:**
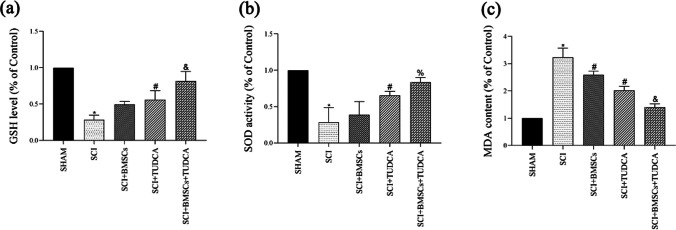
The combined treatment of TUDCA and BMSC transplantation decreased oxidative stress in a rat SCI model. **a** GSH levels in the indicated groups at 10 days post-SCI. **b** The activity of SOD in the indicated groups at 10 days post-SCI. **c** MDA content in the indicated groups at 10 days post-SCI. Data are presented as mean ± SD. ^*^P < 0.05 vs. the SHAM group. ^#^*P* < 0:05 vs. the SCI group. ^&^*P* < 0.05 vs. the BMSC group (*n* = 6)

In rats with SCI, plasma levels of GSH and SOD were reduced, while the MDA level was increased. However, TUDCA combined with BMSC transplantation significantly reversed these changes, indicating a reduction in OS in SCI by TUDCA treatment, either alone or in combination with BMSCs (Fig. 7).

### Effects of BMSCs, TUDCA, and Their Combination on the Nrf-2 Signaling Pathway in SCI

**Fig. 8:**
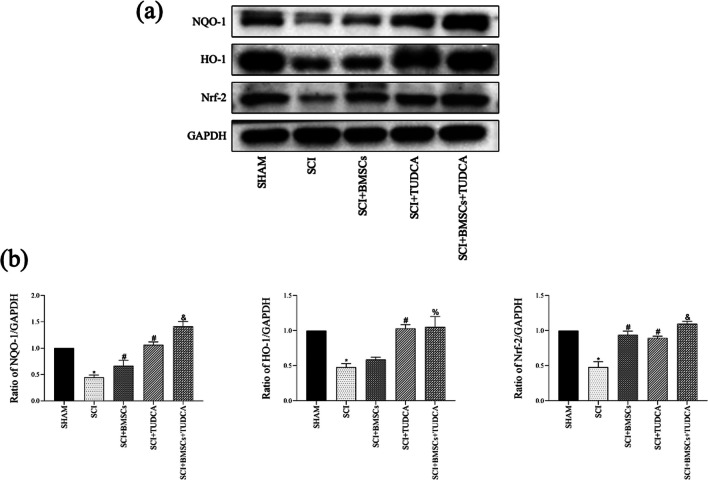
**a**, **b** Western blot and the relative quantification of Nrf-2 signaling pathway–related proteins at 10 days after SCI showing the combined treatment of TUDCA and BMSC transplantation affected the Nrf-2 signaling pathway after SCI. Data are presented as mean ± SD. ^*^P < 0.05 vs. the SHAM group. ^#^*P* < 0:05 vs. the SCI group. ^&^*P* < 0.05 vs. the BMSC group (*n* = 6)

The impact of the Nrf-2 signaling pathway on mediating the combined effects of BMSCs and TUDCA on SCI was assessed by measuring Nrf-2, NQO-1, and HO-1 protein levels for spinal tissue samples. The Nrf-2 signaling pathway is known for its pivotal role in regulating the antioxidant response of cells. Our results showed that the levels of Nrf-2, NQO-1, and HO-1 were increased with statistical significance following TUDCA treatment alone or in combination with BMSCs at 10 days post-SCI, suggesting that the protective impact of BMSCs and TUDCA on SCI may be mediated via the Nrf-2 signaling pathway (Fig. 8).

## Discussion

SCI is an irreversible nerve injury that often results in sensory and motor dysfunction, for which effective solutions have been currently unavailable [26, 27]. Transplantation of cells, especially BMSCs, has emerged as a potential treatment option for SCI [12, 28, 29]. Numerous studies have shown that BMSCs can differentiate into neuron-like cells, promoting neuronal regeneration and angiogenesis while inhibiting inflammation, making them promising candidates for SCI therapy [30–33]. However, BMSC transplantation faces challenges such as low survival rates and limited differentiation potential, primarily due to the OS microenvironment induced by secondary SCI, which significantly hampers treatment effectiveness. Therefore, an effective and timely intervention aimed at alleviating secondary injury can potentially limit nerve damage and facilitate better functional recovery [9, 34, 35]. Previous studies have demonstrated that medication can prolong the survival and enhance the antioxidant capacity of BMSCs within the SCI microenvironment [25, 36, 37]. This study demonstrated that TUDCA significantly increased BMSC viability, decreased apoptosis and OS in vitro and in vivo, accelerated tissue regeneration, reduced GFAP, and facilitated functional recovery following BMSC transplantation in SCI. Moreover, these effects may be induced by the Nrf-2 signaling, as evidenced by Nrf-2, NQO-1, and HO-1 expression. Collectively, these findings suggest that TUDCA exerts antiapoptotic and antioxidant effects, thereby improving the microenvironment both in vitro and in vivo.

TUDCA, the primary active compound found in natural bear bile, has demonstrated neuroprotective, anti-apoptotic, and antioxidant effects in various models of neurological disorders [38–40]. Through the activated Nrf-2/HO-1 pathway, TUDCA inhibits oxidative stress, inflammation, and apoptosis, thereby protecting primary cortical neurons and improving functional recovery [20, 41]. When transplanted into SCI rats, it promotes the formation of anti-neuroinflammatory M2 macrophages and enhances motor function recovery [24]. The above findings demonstrated the potential of TUDCA as one of the SCI treatments. However, despite the promising effects of TUDCA in SCI, it remains unclear whether its combination with BMSC transplantation can provide additional therapeutic benefits. Thus, this study was designed to investigate the underlying mechanisms using rats transplanted with BMSCs, with or without TUDCA. In vitro experiments demonstrated that TUDCA effectively ameliorates cell death, apoptosis, and oxidative injury induced by H_2_O_2_ among BMSCs. Furthermore, in rats treated with both TUDCA and BMSC transplantation, improved gait and locomotive performance were observed, indicating restoration of motor function following SCI. Activation of the Nrf-2 signaling pathway was identified as one possible mechanism through which TUDCA reduced cell apoptosis and OS. However, a previous study reported no additional effect of combined treatment with TUDCA and BMSC compared to BMSC treatment alone, based solely on the BBB score [42]. It is worth noting that the use of different intervention methods and concentrations may have contributed to varying results. Therefore, future studies should consider employing alternative methods and technologies to further investigate it.

Apoptosis exerts a pivotal effect on the secondary injury following primary SCI. Both human and animal studies have demonstrated that neuronal and glial cell death during this phase is often a result of secondary apoptosis [43, 44]. The Bcl-2 family is known to modulate apoptosis, which is an important process in secondary SCI [45]. However, transplanted BMSCs often have poor survival rates within the injured spinal cord, leading to reduced treatment efficacy. In our current study, we utilized an H_2_O_2_-induced BMSC model, which exhibited upregulated Bax level and downregulated Bcl-2 level, indicating increased apoptosis. Remarkably, TUDCA treatment significantly attenuated H_2_O_2_-induced cell death, as confirmed by Annexin V/PI staining. Similarly, in the rat SCI model, we observed consistent results. Furthermore, TUNEL staining validated the antiapoptotic effects of TUDCA in both cellular and animal models, highlighting its potential as an antiapoptotic agent.

SCI can induce an evident increase in OS and free radicals [46], which are major contributors to secondary damage and can hamper the reparative ability of BMSCs. Therefore, strategies that aim to suppress OS have been proposed as viable approaches for successful BMSC transplantation in SCI. OS is characterized by the imbalanced generation of ROS or other oxidants, as well as the body’s capacity to counteract them through antioxidants, such as GSH and SOD, ultimately leading to cellular dysfunction or death [47]. Previous studies [7, 20], consistent with our findings, have reported that TUDCA can enhance CV by inhibiting ROS and MDA production, thereby restoring SOD activity in vitro. In our investigation, both TUDCA treatment alone and the combined therapy demonstrated a reversal of reduced SOD and GSH activity when compared to the SCI and BMSC groups. Additionally, these interventions were found to effectively promote motor functional recovery following SCI. Moreover, both the TUDCA-treated group and the combined therapy group exhibited a reduction in the lesion area as well as an increase in the neuron number displaying normal morphology or Nissl bodies. Furthermore, the neuro-related protein expressions (MAP2, MBP, and TUJ1) were increased, while the protein expression associated with GFAP was reduced in the combination groups. Thus, the observed neuroprotective effects of TUDCA could be attributed to its ability to mitigate OS and cell apoptosis following SCI.

To further understand the mechanism underlying the antioxidant and antiapoptotic effects of TUDCA, we investigated the involvement of the Nrf-2 signaling pathway. Nrf-2, a transcription factor, plays a critical role in regulating various antioxidant enzymes [48]. Normally, Nrf-2 is sequestered in the cytoplasm and remains inactive due to its association with Keap1. Once the cells were exposed to oxidative stress, the sequestration of Nrf-2 by Keap1 would be disrupted, thereby causing the translocation into the nucleus, in which it binds to antioxidant response elements, trigging the transcription of key antioxidant enzymes like HO-1 as well as NQO-1. Activation of this pathway by TUDCA can help protect against cellular OS [49–51]. Both HO-1 and NQO-1 are antioxidant enzymes that play crucial roles in reducing OS following SCI. HO-1 exhibits neuroprotective effects and facilitates neuronal repair, ultimately leading to improved functional recovery after SCI [44, 52, 53]. NQO-1 is a flavoprotein present in all eukaryotic cells and functions as a protective agent by reducing OS and acting as an antioxidant [54]. Our study revealed a notable reduction in the Nrf-2, NQO-1, and HO-1 protein levels in both the BMSC death model induced by H_2_O_2_ and the rat SCI model, which was in accordance with the previously reported results [25, 45].

To investigate how the Nrf-2 signaling pathway mediated the neuroprotective effects induced by TUDCA, we employed Brusatol, a potent inhibitor of this pathway. Brusatol functions by reducing Nrf-2 protein levels through the promotion of its ubiquitination and subsequent degradation, thereby inhibiting the expression of antioxidant enzymes [55]. When TUDCA and Brusatol were simultaneously administered to BMSCs, the protective effects of TUDCA on CV and antioxidant enzyme levels were significantly diminished, suggesting that the Nrf-2 signaling pathway is indeed related to TUDCA’s protective effect. However, the exact mechanisms underlying TUDCA-mediated protection in vivo remain partially understood. Further investigations utilizing Nrf-2 knockout/knock-in models may provide additional evidence to support the significant role of the Nrf-2 signaling pathway in facilitating TUDCA’s antioxidant and antiapoptotic effects.

There were some limitations in our current study. Firstly, the detailed mechanisms by which TUDCA protects BMSCs from H_2_O_2_-induced cytotoxicity remain unclear since we just analyzed some gene expression within the Nrf-2 signaling pathway. Secondly, our study did not involve the use of gene knockout or transgenic mice in vivo. More research should be carried out to solve these limitations in the near future.

## Conclusion

**Fig. 9:**
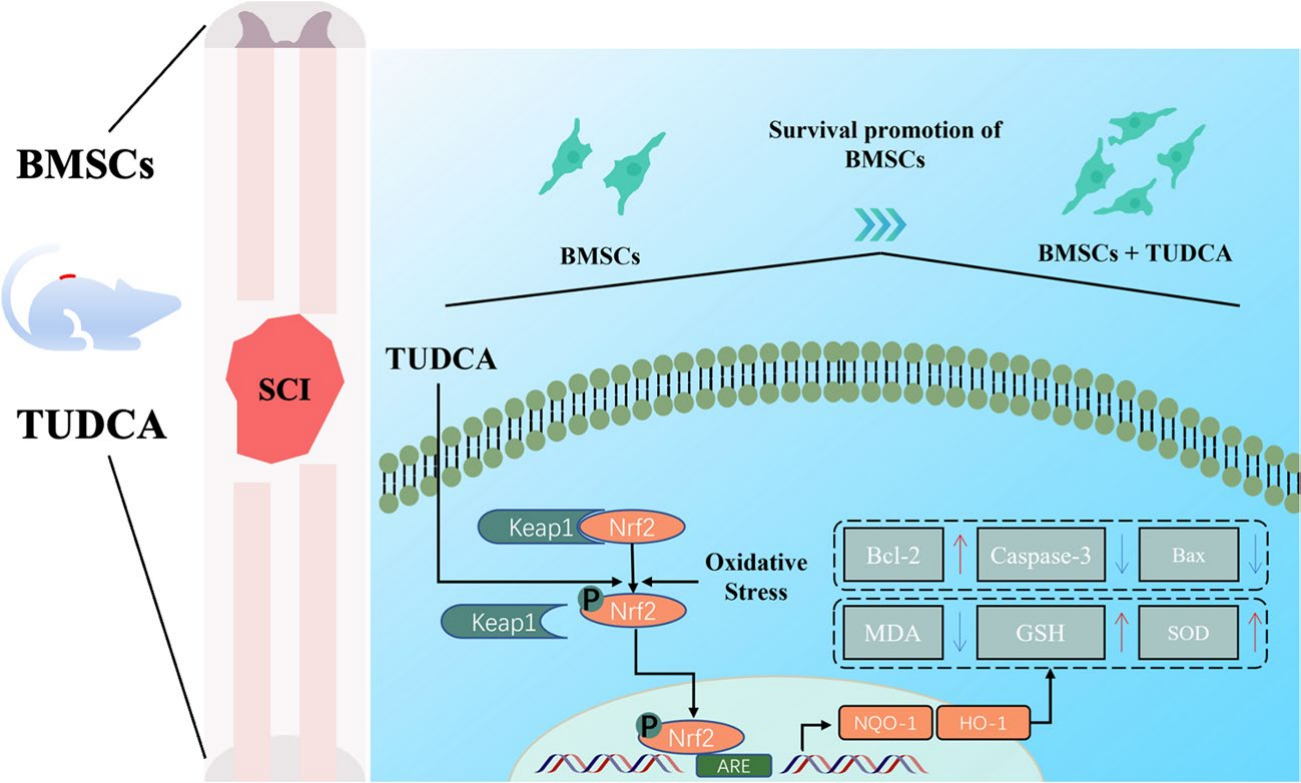
A schematic diagram

The findings in this study indicate that TUDCA may have the potential to protect BMSCs from H_2_O_2_-induced cytotoxicity via the activated Nrf-2 signaling pathway. This pathway may possess antiapoptotic and antioxidant effects, as depicted in Fig. 9. These results underscore the promising therapeutic advantages of combining TUDCA with BMSC transplantation in SCI treatment. The combined therapy may enhance the survival and functionality of transplanted BMSCs. Therefore, TUDCA could serve as a valuable adjunct to BMSC transplantation therapy for SCI, thereby potentially augmenting its therapeutic efficacy.

## Data Availability

The data used to support the findings of this study are available from the corresponding author upon request.

## Abbreviations

DAPI: 4′,6-Diamidino-2-phenylindole
BBB: Basso, Beattie, and Bresnahan
BMSC: Bone marrow mesenchymal stem cell
CCK-8: Cell counting kit-8
DCFH-DA: Dichloro-dihydro-fluorescein diacetate
GFAP: Glial scar formation
HO-1: Heme oxygenase-1
MAP2: Microtubule-associated protein 2
MBP: Myelin basic protein
Nrf-2: Nuclear factor E2-related factor 2
NQO-1: NAD(P)H: quinone oxidoreductase-1
OS: Oxidative stress
PBS: Phosphate-buffered saline
ROS: Reactive oxygen species
SCI: Spinal cord injury
SD: Sprague Dawley
TUDCA: Tauroursodeoxycholic acid
TUJ1: Beta-tubulin III
H&E: Hematoxylin and eosin
