# Correction: Tauroursodeoxycholic Acid Inhibited Apoptosis and Oxidative Stress in H2O2-Induced BMSC Death via Modulating the Nrf-2 Signaling Pathway: the Therapeutic Implications in a Rat Model of Spinal Cord Injury

**DOI:** 10.1007/s12035-024-04236-y

**Published:** 2024-05-23

**Authors:** Jiaxian Weng, Le Wang, Kai Wang, Haitao Su, Dan Luo, Haimei Yang, Yaqian Wen, Qiduan Wu, Xing Li

**Affiliations:** 1 School of Pharmaceutical Sciences, Guangzhou University of Chinese Medicine, Guangzhou, 510006 Guangdong China; 2 Lingnan Medical Research Center of Guangzhou University of Chinese Medicine, Guangzhou, 510405 China; 3 Guangzhou University of Chinese Medicine, Guangzhou, 510405 China; 4 Department of Spine Surgery, the First Affiliated Hospital of Sun Yat-Sen University, Guangdong Provincial Key Laboratory of Orthopedics and Traumatology, Guangzhou, 510080 Guangdong China; 5 State Key Laboratory of Dampness Syndrome of Chinese Medicine, Department of Orthopedic Surgery, The Second Affiliated Hospital of Guangzhou University of Chinese Medicine, Guangzhou, 510120 China; 6 The First Affiliated Hospital of Chinese Medicine, Guangzhou University of Chinese Medicine, Guangzhou, 510405 China

**Correction: Molecular Neurobiology**

10.1007/s12035-023-03754-5

The original version of this article unfortunately contained an error, specifically in the author's affiliation assignment. The author Xing Li affiliation is incorrectly assigned. He is only affiliated to "State Key Laboratory of Dampness Syndrome of Chinese Medicine, Department of Orthopedic Surgery, The Second Affiliated Hospital of Guangzhou University of Chinese Medicine, Guangzhou 510120, China". Thus in author list, it should be listed as Xing Li5 instead of Xing Li1,3,5.

The original article has been corrected.

